# Remodeling of the Residual Gastric Mucosa after Roux-En-Y Gastric Bypass or Vertical Sleeve Gastrectomy in Diet-Induced Obese Rats

**DOI:** 10.1371/journal.pone.0121414

**Published:** 2015-03-30

**Authors:** Konstantinos Arapis, Jean Baptiste Cavin, Laura Gillard, Françoise Cluzeaud, Philippe Lettéron, Robert Ducroc, Johanne Le Beyec, Muriel Hourseau, Anne Couvelard, Jean-Pierre Marmuse, Maude Le Gall, André Bado

**Affiliations:** 1 Inserm UMR 1149, UFR de Médecine Paris Diderot, Université Paris Diderot, Sorbonne Paris Cité, Paris, France; 2 DHU Unity, Bichat-Beaujon AP-HP, Paris, France; 3 Service de Chirurgie Générale et Digestive; Hôpital Bichat—Claude Bernard. Paris, France; 4 Département de Pathologie; Hôpital Bichat—Claude Bernard, Paris, France; INRA, FRANCE

## Abstract

Whereas the remodeling of intestinal mucosa after bariatric surgeries has been the matter of numerous studies to our knowledge, very few reported on the remodeling of the residual gastric mucosa. In this study, we analyzed remodeling of gastric mucosa after Roux-en-Y gastric bypass (RYGB) and vertical sleeve gastrectomy (VSG) in rats. Diet-induced obese rats were subjected to RYGB, VSG or sham surgical procedures. All animals were assessed for food intake, body-weight, fasting blood, metabolites and hormones profiling, as well as insulin and glucose tolerance tests before and up to 5 weeks post-surgery. Remodeling of gastric tissues was analyzed by routine histology and immunohistochemistry studies, and qRT-PCR analyses of ghrelin and gastrin mRNA levels. In obese rats with impaired glucose tolerance, VSG and RYGB caused substantial weight loss and rats greatly improved their oral glucose tolerance. The remaining gastric mucosa after VSG and gastric pouch (GP) after RYGB revealed a hyperplasia of the mucous neck cells that displayed a strong immunoreactivity for parietal cell H^+^/K^+^-ATPase. Ghrelin mRNA levels were reduced by 2-fold in remaining fundic mucosa after VSG and 10-fold in GP after RYGB. In the antrum, gastrin mRNA levels were reduced after VSG in line with the reduced number of gastrin positive cells. This study reports novel and important observations dealing with the remaining gastric mucosa after RYGB and VSG. The data demonstrate, for the first time, a hyperplasia of the mucous neck cells, a transit cell population of the stomach bearing differentiating capacities into zymogenic and peptic cells.

## Introduction

The obesity epidemic has grown in severity over the past several decades and is now a worldwide public health priority. Obesity induces various lifestyle-related diseases, such as type 2 diabetes (T2D), hyperlipidemia, hypertension, and fatty liver disease, and is causing a major health problem in terms of morbidity and mortality. Bariatric surgical procedures are currently accepted as the most effective treatment for morbid obesity [1–4]. Besides weight loss, bariatric surgery also provides the possibility of resolving or improving several comorbidities [5–7]. Two major gastrointestinal weight-loss surgeries are currently used: the vertical sleeve gastrectomy (VSG) and the Roux-en-Y gastric bypass (RYGB). VSG provides reduction of gastric volume through resection of the majority of the corpus of the stomach along the greater gastric curvature and construction of a tubular gastric pouch [8]. In RYGB, a small gastric pouch (GP) is also created but in addition the jejunum is transected, and the distal portion of the small intestine (mid-jejunum and ileum) is connected directly to the GP so that meal contents bypass the lower stomach and the upper small bowel [9,10]. The duodenal-upper jejunal segment is anastomosed at a distal site in the jejunum so that gastric, hepato-biliary and exocrine pancreatic drainage contacts luminal nutrients only in the latter half of their passage through the gut. Along with sustained weight loss, VSG and RYGB lead to rapid and significant improvement or resolution of comorbid disease states, especially T2D [11]. The improvements of insulin sensitivity and glucose tolerance are independent of weight loss in rodents and humans [4]. Changes in incretin secretion, such as increased glucagon-like peptide-1 (GLP-1), play an important role, but the exact biochemical mechanisms are still not fully understood [10–14] and even the importance of GLP-1 has recently been challenged[15,16]. Thus, further research on the effect of bariatric surgery on metabolic state beyond weight loss is required.

The fundamental anatomic difference between the two procedures is that, in VSG only the anatomy of the stomach is changed and there is no reconfiguration of the intestine. In RYGB, the entero-insular axis as well as hepato-portal sensing of nutrients are drastically changed. In addition, RYGB may involve disruption of vagal fibres whereas VSG certainly does not. The maintenance of specific vagal fibres, *e*.*g*. dorsal neurovascular bundle or celiac branch, are important for at least some of RYGB-induced effects [17,18] whereas others, *e*.*g*. hepatic branch may be of less importance [19]. Therefore, comparing these two surgical procedures provide a unique opportunity to study the ways by which different parts of the gastrointestinal (GI) tract contribute to the regulation of physiological processes, such as the regulation of body weight, food intake and improvement of glucose metabolism.

The increasing demand for understanding how bariatric procedures are effective has led to the development of animal models of surgery [20,21]. Although data arising from animal models may not be suitable for direct extrapolation to humans, they allow the investigation of factors that are impossible to be evaluated in individuals. Whereas the remodeling of intestinal mucosa after gastrointestinal weight-loss surgeries has been the matter of numerous studies [22–25] to our knowledge, only few reported the remodeling of the remaining stomach mucosa after VSG or RYGB [26].

In this study, we developed RYGB and VSG surgeries in HFD-fed rats in order to monitor changes in body composition, food intake, glucose tolerance and analyzed remodeling of the remnant gastric mucosa.

## Materials and Methods

### Animals and diets

All animal use conformed to the European Community guidelines and was approved by the local ethics committee (*N° #2011-14/773-0030* Comité d'Ethique Paris-Nord) and the Ministry of Higher Education and Research (*N° 02285*.*01)*.

Male Wistar rats weighing 220–240g (Janvier Labs, Le Genest-St-Isle, France) were caged under standard laboratory conditions with tap water and food provided *ad libitum*, in a 12h/12h light/dark cycle at temperature of 21–23°C. The rats were allowed to acclimate for one week before being subjected to any experimental procedure. They were fed normal chow (ND) or high-fat diet (HFD) (1320 and C1090-45, respectively Altromin, Genestil, Royaucourt, France) for 12 weeks before surgery.

### Surgical Procedures

**Fig 1:**
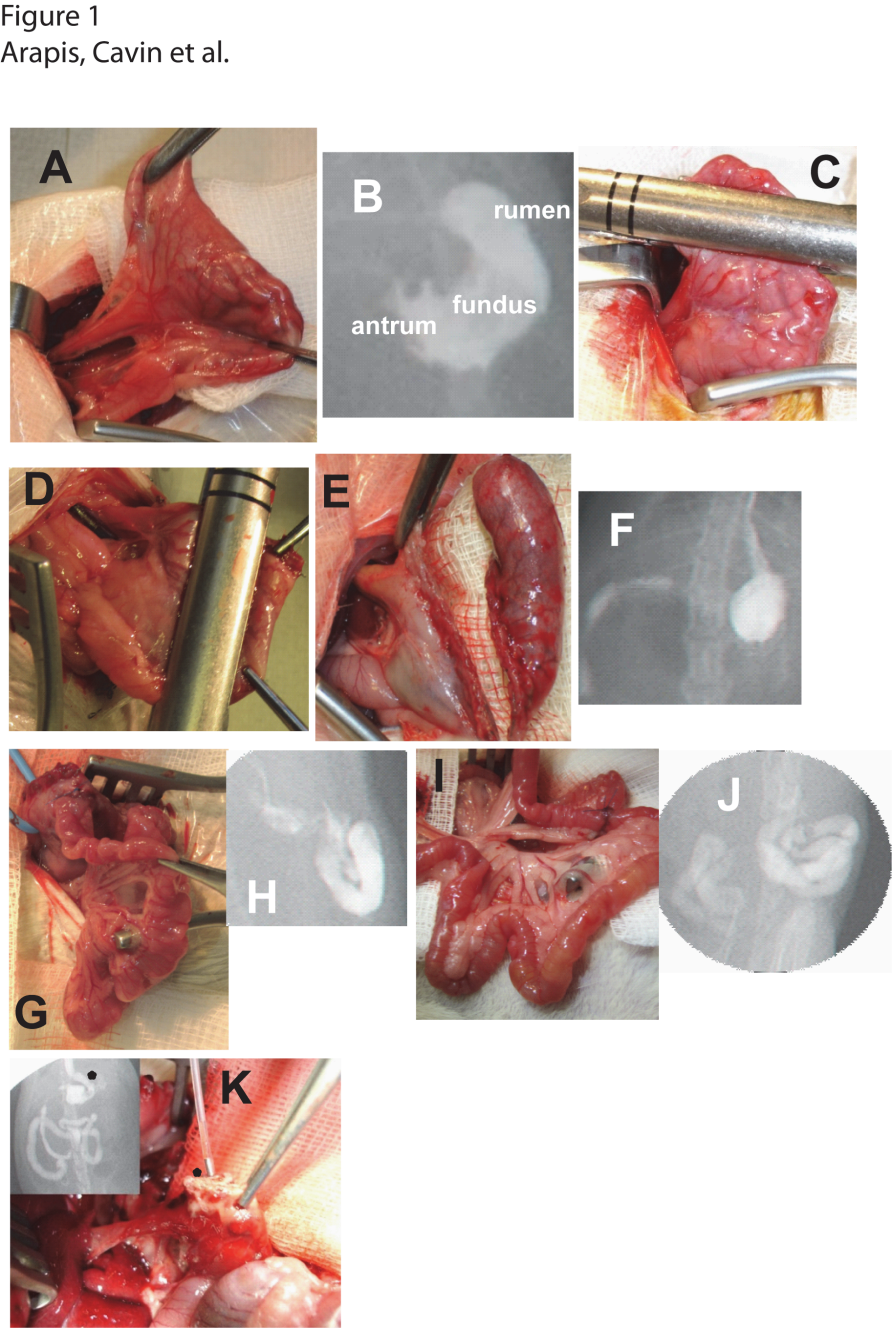
Surgical procedures of Vertical Sleeve Gastrectomy (VSG) and Roux-en-Y Gastric Bypass (RYGB) in rats. ***(A*)** Photography and **(B)** contrast radiography of a rat stomach showing forestomach (rumen), fundus and antrum. In both surgeries, first step is the resection of the rumen **(C)**. In VSG: vertical resection of the fundus **(D, E)** resulted in a reduction of the gastric volume visible by opacification with gastrograffine **(F)**. In RYGB: reconstructed gastric pouch directly anastomosed to the jejunum **(G)** and verification of the gastro-jejunal anastomosis (alimentary limb) after opacification **(H)**. In RYGB: jejuno-jejunal anastomosis limb **(I)** and verification of the gastro-jejunal anastomosis after opacification **(J). (K)** Example of a fistula, the main cause of the post-operative mortality. Photography of the necropsy of one rat showing a fistula *(star)* in the remaining stomach. *Insert*: radiography imaging after opacification with gastrograffine of the gastrointestinal tract showing a fistula *(star)*.

Animals were randomly divided into VSG group, RYGB group and corresponding sham-operated (sham) (S1 Fig.). They were fasted overnight before operation. Anesthesia was induced by intraperitoneal injection of pentobarbital (Ceva, Libourne, France). Standard aseptic procedures were used throughout. After laparotomy, the stomach was isolated outside the abdominal cavity (Fig. 1A). Loose gastric connections to the spleen and liver were released along the greater curvature, and the suspensory ligament supporting the upper fundus was severed. For all surgical procedures, the first step was the resection of the forestomach, a non-glandular part of the stomach (Fig. 1C) by one application of ETS-Flex 35-mm staple gun (Ethicon, Issy les Moulineaux, France).

### Vertical Sleeve Gastrectomy (VSG)

The procedure consists in a 80% resection of gastric fundus stomach by one application of ETS-Flex 35-mm staple gun (Ethicon, Issy les Moulineaux, France), leaving a thin gastric tube in continuity with the oesophagus like in humans and keeping the antrum in place (Fig. 1D and E).

### Roux-en-Y Gastric Bypass (RYGB)

First, the terminal oesophagus and the great curvature were dissected free. After the first step consisting in the resection of the rumen with an ETS blue cartridge as above (Fig. 1C), the lesser curvature was then dissected and the supply vascular was isolated in this region. A smooth tube passed behind the œsophagus for the creation of the gastric pouch with an application of a TA-DST 30 mm-3.5mm (Covidien, New Heaven, US). The retaining pin of the stapler passed through the dissection of the lesser curvature. The instrument was positioned in a parallel line with the transection of the rumen. With this method, we created a gastric pouch (GP) < 5% of the original stomach (Fig. 1G). The jejunum was then transected 15cm distally from the pylorus. The Roux-en-Y limb was created by an end to lateral jejuno-jejunal anastomosis with 6–0 PDS running sutures 20 cm distally to the section (Fig. 1I). The final step was the gastro-jejunal anastomosis with 6–0 PDS running sutures (Fig. 1G). The total bypassed tube was 25 cm which proportionally corresponds to the one frequently created in human tube. The sham procedure involved gastrotomy, enterostomy, and repair.

### Post-operative care

Post-surgery, rats received acute care for 1 to 3 days consisting of twice-daily subcutaneous injections of 10 mL Bionolyte G5 (Sodium chloride (0.4%) glucose (5.5%) Potassium Chloride (0.2%))(Baxter, Maurepas, France) and daily subcutaneous administration of 20,000 units/kg penicillin G (Panpharma, Luitre, France). Rats were given access to a liquid diet (C-0200, Altromin, Genestil, Royaucourt, France) on day 2 to 3 after VSG or on day 3 to 5 after RYGB and the rats were therafter shift to *ad libitum* normal diet.

### Radiography of the gastrointestinal tract before and after surgery

Verification of surgical procedures was performed by contrast radiography of esophageal-gastro intestinal opacification with an oral load of Gastrografine (Bayer Santé, Puteaux, France) followed by radiographic imaging using a MD3system (Phillips, France) (Fig. 1).

### Measurement of food intake, body weight and whole body composition

Body weight and food intake were measured daily and the average daily food intake was calculated taking spillage into account. Whole body composition was measured before and after surgery in un-anesthetized but physically restrained rats using an Echo Medical systems’ EchoMRI 900 (Whole Body Composition Analyzers, EchoMRI, Houston, USA).

### Glucose and insulin tolerance tests

RYGB- VSG- and sham-operated rats were fasted for 16 h before being subjected to an oral glucose tolerance test (OGTT: 1g/kg BW). Blood was sampled from the tail vein before (t = 0) and 5, 15, 30, 60 and 120 min after the administration of glucose. For ITT, non-fasted rats received an intraperitoneal injection of 1U insulin per kg BW. Glucose was measured with the AccuChek System (Roche Diagnostics, Meylan, France) and expressed in mg/dL. Areas under the curves were calculated using the trapezoidal rule with GraphPad Prism (GraphPad Software, San Diego, CA, USA).

### Plasma analysis

Collected blood was used for the determination of glucose, triglycerides (TG), total cholesterol, high-density lipoprotein (HDL)-cholesterol, alanine transaminase (ALT) and aspartate transaminase (AST) activities using an automatic analyzer AU400 (Olympus Diagnostics, Rungis, France). Plasma leptin, insulin, C-peptide, glucagon, tumor necrosis factor (TNF)-alpha, and Monocyte chemotactic protein (MCP)-1 levels were assayed using a multiplex immunoassay kit (Merck Millipore, Saint Quentin en Yvelines, France).

### RNA isolation and RTqPCR

Total RNA was extracted from frozen fundic and antral mucosa scrapings with Trizol reagent (Life Technologies, Courtaboeuf, France) and cDNA were generated and quantified as previously reported [27] with the Light Cycler System (Roche Diagnostics, Indianapolis, IN, USA) according to the manufacturer's instructions and using specific primers (S1 Table).

### Immunohistochemical studies

Stomach segments were fixed overnight in 10% neutral buffered formalin, paraffin-embedded, and sectioned at 5 μm. The sections were stained with hematoxylin and eosin or with Periodic acid-Schiff/Alcian blue to detect the presence of neutral (pink) and acidic (blue) mucins respectively. For immunohistochemistry, after antigen retrieval, the sections were immunolabelled with rat monoclonal anti-Ki67 antibody (DAKO, Les Ulis, France), mouse monoclonal anti-H^+^/K^+^ ATPase (β Subunit) antibody (Sigma Aldrich, Saint-Quentin Fallavier, France), with rabbit polyclonal anti-gastrin antibody (Immunostar, Hudson, WI) using a detection kit (Bond Polymer Refine detection; DS9800; Leica Microsystems). Substitution of the primary antibody with PBS was used as a negative control. Immunofluorescence of gastrin was also performed using an Alexa Fluor 488 goat anti-rabbit as secondary antibody. Immunostainings were evaluated by two (MH, AC) investigators from the department of Pathology, blinded to the slides. Each slide was scanned with Aperio ScanScope CS System (Leica Microsystemes SAS, Nanterre France) and the images were analyzed with TRIBVN CaloPix software (TRIBVN, Chatillon France) by the Imaging Specialist (V. Descatoire) from the department of pathology.

### Statistical analysis

All values are expressed as means ± S.E.M. All the statistics used non parametric tests, Mann Whitney to compare two groups and Kruskall Wallis and Dunns post hoc tests to compare more than two groups. Statistical analyses were performed with GraphPad Prism version 5.0 for Windows (GraphPad Software, SanDiego, CA, USA). A value of P < 0.05 was considered to be statistically significant.

## Results

### High-Fat Diet induces obesity and glucose intolerance in rats

As expected, rats, fed with HFD for 3 months, exhibited significantly enhanced body weight (+21%, P<0.01 *vs*. ND), that was essentially due to increase in fat mass (+ 93%, P<0.01 *vs*. ND) associated with reduction in percentage of lean mass (-12.5%, P<0.01 *vs*. ND) whereas total lean mass slightly increased (394.4 ± 29 for ND *vs*. 411 ± 24g for HFD, NS) (S2A-C Fig.). Circulating levels of TG were increased whereas HDL and total cholesterol were not significantly different (Table 1). Fasted blood glucose levels were increased in 3-month HFD *vs*. ND rats (Table 1). Oral glucose (OGTT) and insulin (ITT) tolerance tests (S2D–E Fig.) were worsened in HFD rats (*vs*. ND rats). Thus, 3 months after HFD feeding, the rats became obese and exhibited oral glucose intolerance and insulin resistance exhibiting a prediabetic status. Interestingly at that time, 30% of the obese rats showed signs of hepatic steatosis and exhibited increased hepatic FAS and ACC mRNA levels (S2F–H Fig.). In summary, our model of diet-induced obese rats appears close to that found in the common human obesities associated with metabolic syndrome.

**Table 1:** Biochemical parameters of operated rats. Biochemical parameters of 6–8 week old male Wistar rats fed 3-months with ND or HFD before and 2 weeks after vertical sleeve gastrectomy (VSG) and Roux-en-Y Gastric Bypass (RYGB). Results are the means ± SEM of at least n = 4 in each group.

	3-month ND	3-month HFD		
		before surgery	2 Wks post-VSG	2 Wks post-RYGB
Blood glucose (mg/dL)	82.47± 1.35	99.88 ± 1.94^a^	108 ± 1.53	93.75 ± 5.8
Triglyceridemia (mmol/L)	1.22 ± 0.1	1.76 ± 0.12^a^	0.96 ± 0.14^b^ ^c^	0.53 ± 0.06^c^
HDL-cholesterol (mmol/L)	1.33 ± 0.28	1.27 ± 0.06	1.17 ± 0.07	1.27 ± 006
Cholesterol (mmol/L)	2.58 ± 0.62	2.31 ± 0.73	2.46 ± 0.19	2.41 ± 0.12
Non-esterified fatty acid (mmol/L)	1.69 ± 0.17	1.32 ± 0.08	1.41 ± 0.18^b^	0.68 ± 0.05^c^
Albumin (g/L)	33.58 ± 2.34	33.9 ± 0.5	28.3 ± 0.76	26.3 ± 0.34^c^
ALT (UI)	56.73 ± 13.06	56.0 ± 5.39	64.0 ± 26.1	106.2 ± 37.4
AST (UI)	105.1 ± 26.22	107.0 ± 6.3	140 ± 36.3	175.6 ± 54.7

^a^ P<0.05 ND *vs* HFD;

^b^ P<0.05 RYGB *vs* VSG;

^c^ P<0.001 *vs* before surgery.

The HFD obese rats underwent the VSG or RYGB surgical procedure (Fig. 1) with a perioperative mortality of 12% and 25%, respectively. The cause of death in VSG group was fistula in more than 75% of cases (Fig. 1K), and those in RYGB group were fistula (50%), anastomotic leak (35%) or respiratory complications (15%).

### Food intake and weight loss after VSG and RYGB in obese and lean rats

**Fig 2:**
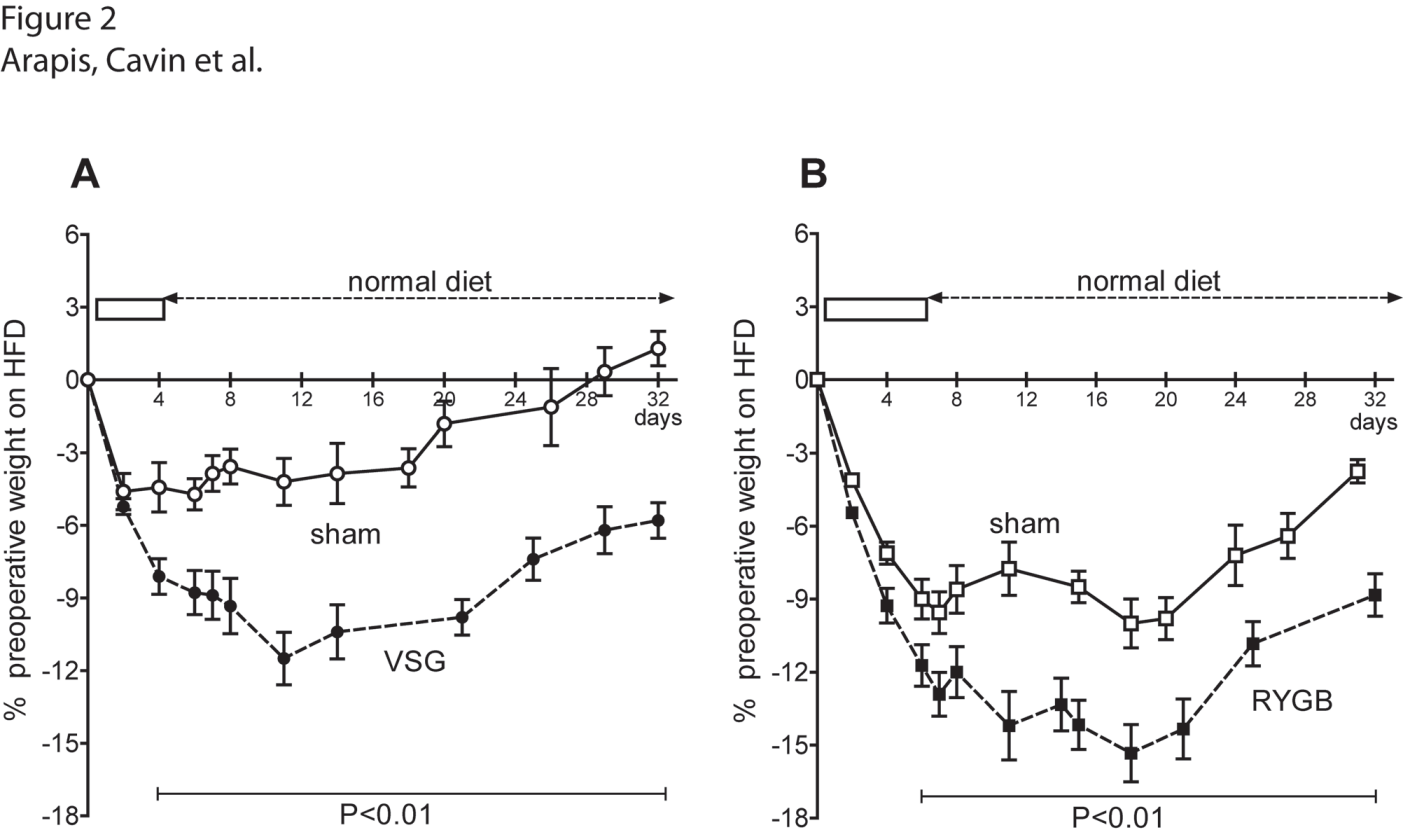
Time-course of body weight after VSG and RYGB in HFD obese rats. **(A)** VSG- and **(B)** RYGB-induced weight loss in HFD obese rats and in the corresponding sham-operated rats. *Black boxes* correspond to the period of post-operative care and liquid diet consumption before the animals had free access to solid ND. Results are expressed as percent of loss of body weight over preoperative weight. Two-Way ANOVA was used to compare body-weight curves.

VSG animals lost up to 11% of their body weight at day 12 post-surgery (11±1.3%, P<0.01 *vs* sham) and then progressively gained weight without reaching their preoperative weight 5 weeks after surgery (Fig. 2A) whereas sham rats did. Food intakes in both groups at that late time point were similar (81.05 ± 2.9 kCal/day for VSG *vs*.75.3 ± 4.65 kCal/day for sham, NS). RYGB animals lost significantly more body weight (-14.2% *vs*. -7.7% in sham at day 12 post-surgery; P<0.01) and still displayed significant weight lost compared to sham rats after 5 weeks (Fig. 2B) whereas food intake was similar in both groups (81.9 ± 3.97 kCal/day for RYGB *vs*. 82.3 ± 6.1 kcal/day for sham; NS). To test whether the switch from HFD to ND in the postoperative diet was determinant or not in the observed effects, we combined similar surgeries on ND fed rats preoperatively **(** S3 Fig.). As VSG and RYGB remain efficient to reduce weight gain in ND fed rats, this indicates that food is not solely responsible for weight loss in HFD rats and that VSG and RYGB surgeries also contribute to the effect.

Finally, quantification of two inflammation markers; TNFalpha and MCP-1 revealed no significant differences in their circulating levels 2 weeks after surgery between sham- and bariatric-operated animals (S4A–B Fig.).

### RYGB and VSG on HFD obese rats improve oral glucose tolerance

**Fig 3:**
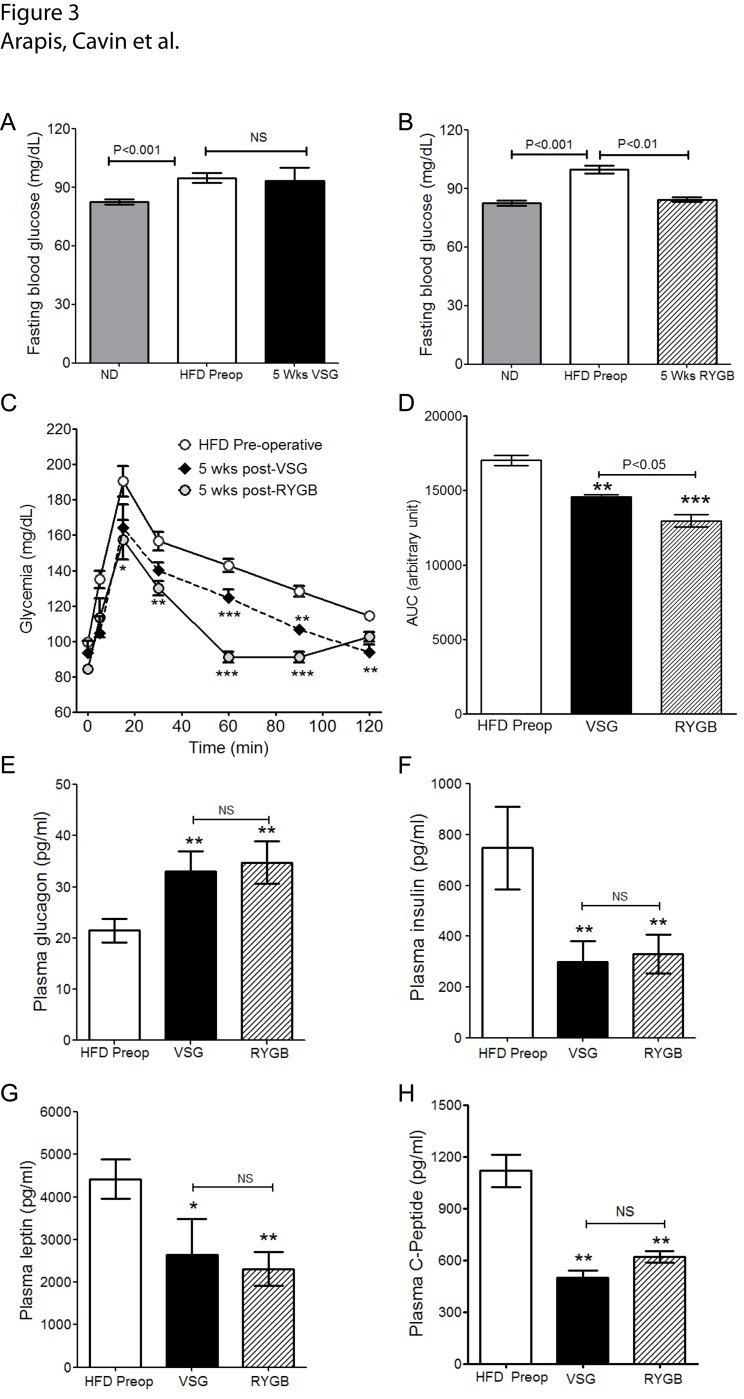
RYGB and VSG improve oral glucose tolerance. **(A-B)** Fasting glucose levels of HFD rats before (HFD Preop) and 5 weeks after VSG **(A)** or RYGB **(B)** in comparison to ND fed rats (ND). **(C)** Comparative time-dependent blood glucose levels after an oral load of 1g/kg BW glucose and **(D)** the corresponding calculated area under curves (AUC) in HFD-fed rats before (HFD Preop) and 5 weeks after VSG or RYGB (n = 12 for HFD, n = 8 for VSG or RYGB). ** P<0.01 and ***P<0.001 *vs*. HFD Preop. **(E-H)** Plasma levels of metabolic hormones in HFD-fed rats before surgery (pre-operative) and 5 weeks after VSG or RYGB surgery **(E)** Glucagon, **(F)** insulin, **(G)** leptin and, **(H)** C-peptide levels. n = 12 pre-operative, n = 6 VSG and n = 6 RYGB.* P<0.05; ** P<0.01 *vs*. HFD Preop: NS: Not significantly different between RYGB and VSG.

After five weeks, VSG was not associated with significant changes in fasting blood glucose levels, whereas RYGB exhibited a 15% decrease (P<0.05 *vs*. before surgery) (Fig. 3A and B, Table 1).

OGTT were performed before surgery and 5 weeks after surgery to assess impact on glucose tolerance. Five weeks after surgery, RYGB and VSG rats exhibited an improvement of oral glucose tolerance (Fig. 3C) reflected by reduction in the AUC of 15% for VSG (P<0.01; *vs*. before) and 24% for RYGB (P<0.001; *vs*. before) (Fig. 3D).

We next determined the plasma levels of key pancreatic hormones controlling glucose homeostasis. We found that, 5 weeks post-surgery, at the time of significant improvement of glucose tolerance in RYGB and VSG rats, plasma glucagon increased similarly (Fig. 3E). These changes were associated with a similar decrease in plasma insulin, peptide C and leptin levels (Fig. 3F-H).

### Post-surgical remodeling of gastric mucosa

**Fig 4:**
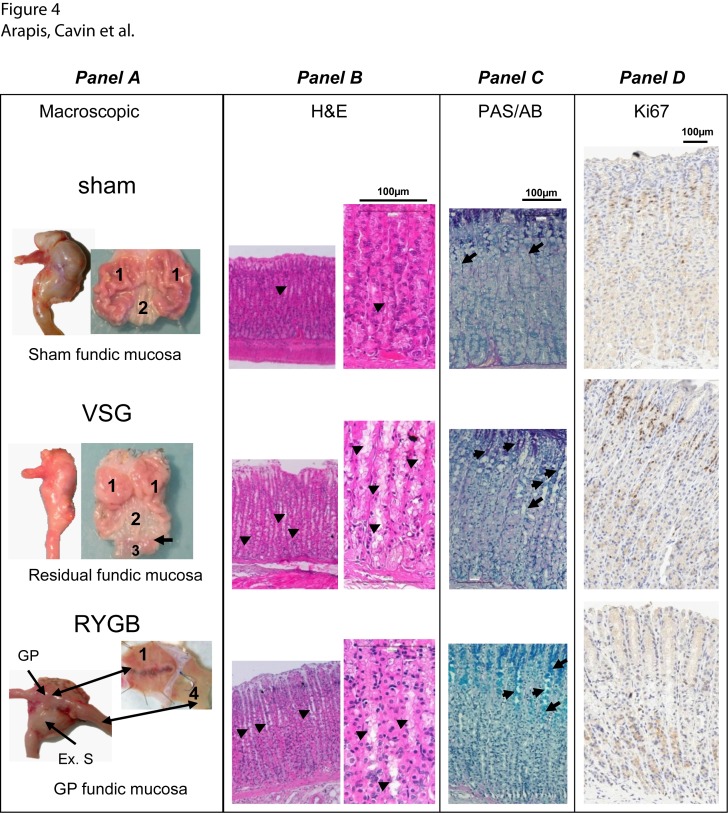
Histology of gastric fundic mucosa in sham-, VSG- and RYGB- operated rats. **Panel A.** Post-mortem representative macroscopic photomicrographs of stomach of sham-, VSG- or RYGB-operated rats. *(top)* normal stomach showing fundus (1) and antrum (2); *(middle)* residual stomach after VSG with fundus (1), antrum (2), duodenum (3) and, pylorus *(arrow)*; *(bottom)* excluded stomach in RYGB and gastric pouch (1) directly anastomosed to the jejunum (4). **Panel B:** representative Hematoxylin and Eosin staining (H&E) of fundic mucosa. Note the hyperplasia of mucous neck cells *(black arrow heads)* after VSG and RYGB. **Panel C:** fundic mucosa sections stained with periodic acid Schiff (PAS)/Alcian blue (AB). Mucous neck cells are PAS/AB positive *(black arrows)*. **Panel D:** immunostaining of Ki67-proliferating cells *(brown nuclei)* in formalin-fixed fundic mucosa section from sham *(top)*, remaining stomach after VSG *(middle)*, and GP after RYGB *(bottom)*.

Five weeks post-surgery, macroscopic examination of the residual stomach in VSG revealed that the antral and fundic surfaces were largely increased **(** Fig. 4, *panel A*). Histological and immunohistochemical analyses of the fundic mucosa from residual gastric VSG and GP in RYGB showed no alteration of the gross architecture and organization of fundic mucosa (Fig. 4, *panel B*). There was no significant increase in the height of fundic mucosa but a hyperplasia of mucous neck cells (MNC) was observed in fundic glands of both VSG and RYGB (Fig. 4, *panel B*). These MNC were further shown to be Periodic Acid-Schiff/Alcian Blue (PAS/AB)-positive cells (Fig. 4, *panel C*) indicating that they have a mucus-secreting phenotype. Moreover quantification of the Ki67 immunostaining of fundic mucosa (Fig. 4, *panel D*) showed no significant change in the percentage of Ki67-positive cells after sleeve (28 ± 2.47 *vs*. 26.2 ± 2.26 Ki67-positive cells per mucosa area, NS *vs*. sham). In GP mucosa after RYGB, no labeling of Ki67 proliferating cells was detected, whereas dense Ki67-positive proliferating cells are detected in the crypts of the jejunum directly anastomosed to the GP indicating that the absence of Ki67 staining was not an artefact (S5 Fig.).

**Fig 5:**
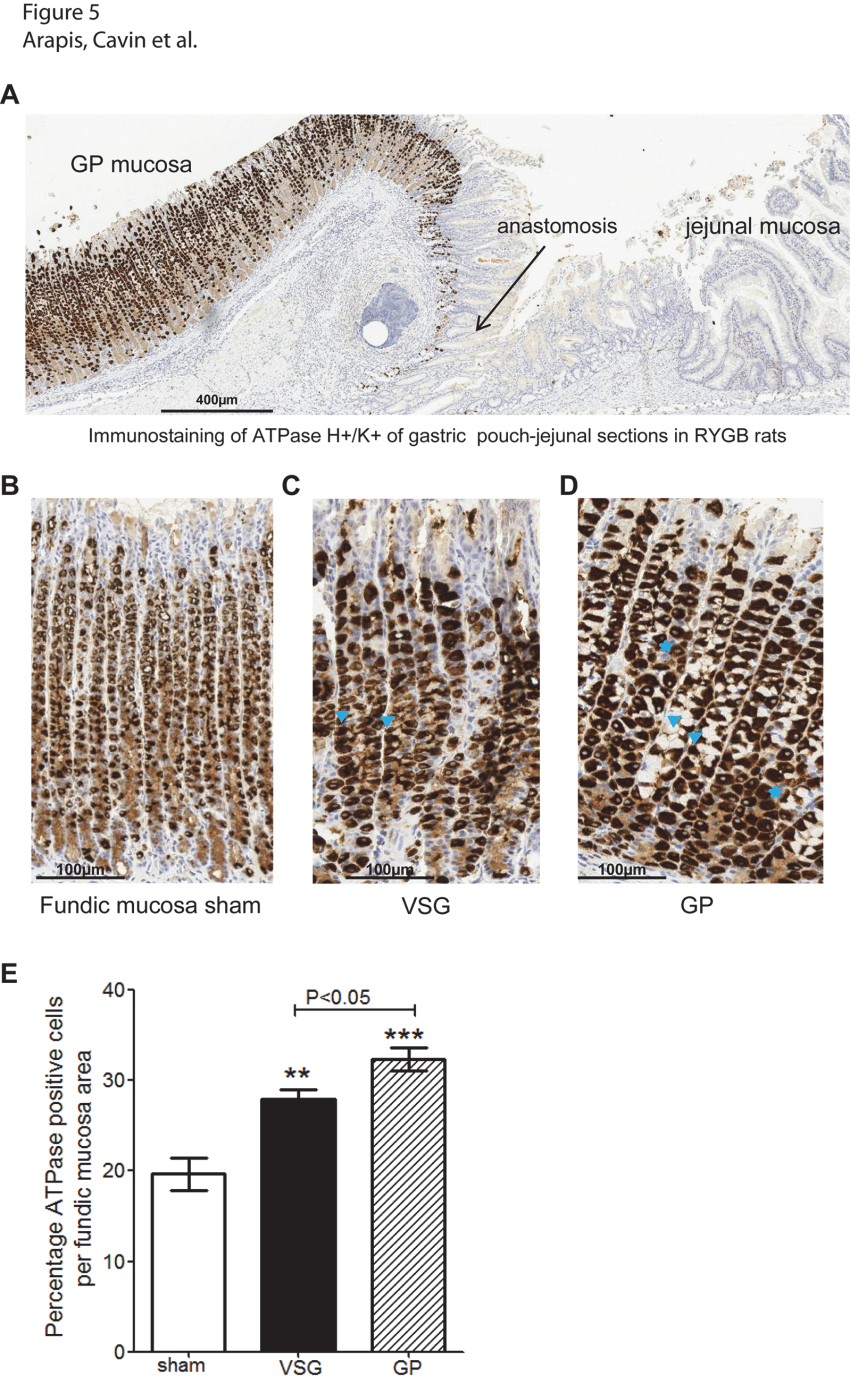
Immunostaining of parietal cell H^+^/K^+^-ATPase in fundic mucosa. **(A)** Overview of a representative immunostaining of parietal cell H+/K^+^-ATPase along the formalin-fixed gastric pouch anastomosed to jejunum in the alimentary limb after RYGB. Comparative immunostaining of parietal cell H+/K^+^-ATPase in fundic mucosa from sham **(B)**, residual stomach after VSG **(C)**, and GP after RYGB **(D)**. Note the enlarge parietal cells *(blue stars)* with dense H^+^/K^+^ATPase immunoreactivity as compared to sham. **(E)** Percent of H^+^/K^+^-ATPase positive cells per fundic mucosa area (μm^2^). Values are shown as mean ± SEM n = 5 for sham and n = 6 for VSG and RYGB. **P<0.01 and ***P<0.001 *vs*. sham.

Furthermore, immunostaining of H^+^/K^+^-ATPase (acid pump) showed dense immunoreactivity in GP mucosa after RYGB whereas no labeling was detected in jejunal mucosa anastomosed to the GP (Fig. 5A). In addition to the dense immunoreactivity of H^+^/K^+^-ATPase, parietal cells also appear larger in fundic mucosa after VSG and in GP after RYGB in comparison to cells in sham fundic mucosa (Fig. 5B-D). Finally, percentage of H^+^/K^+^-ATPase positive cells in the fundic mucosa significantly increased by 40%, (P<0.01 *vs*. sham) and 49% (P<0.001 *vs*.sham) in residual VSG fundic mucosa and in RYGB GP fundic mucosa, respectively (Fig. 5E). These observations are likely to suggest that remaining fundic mucosa after VSG or reconstructed GP after RYGB have almost recovered their acid-secretory capacities despite the initial reduced volume of the stomach corpus.

### Reduction of ghrelin and gastrin mRNA levels after RYGB and VSG

**Fig 6:**
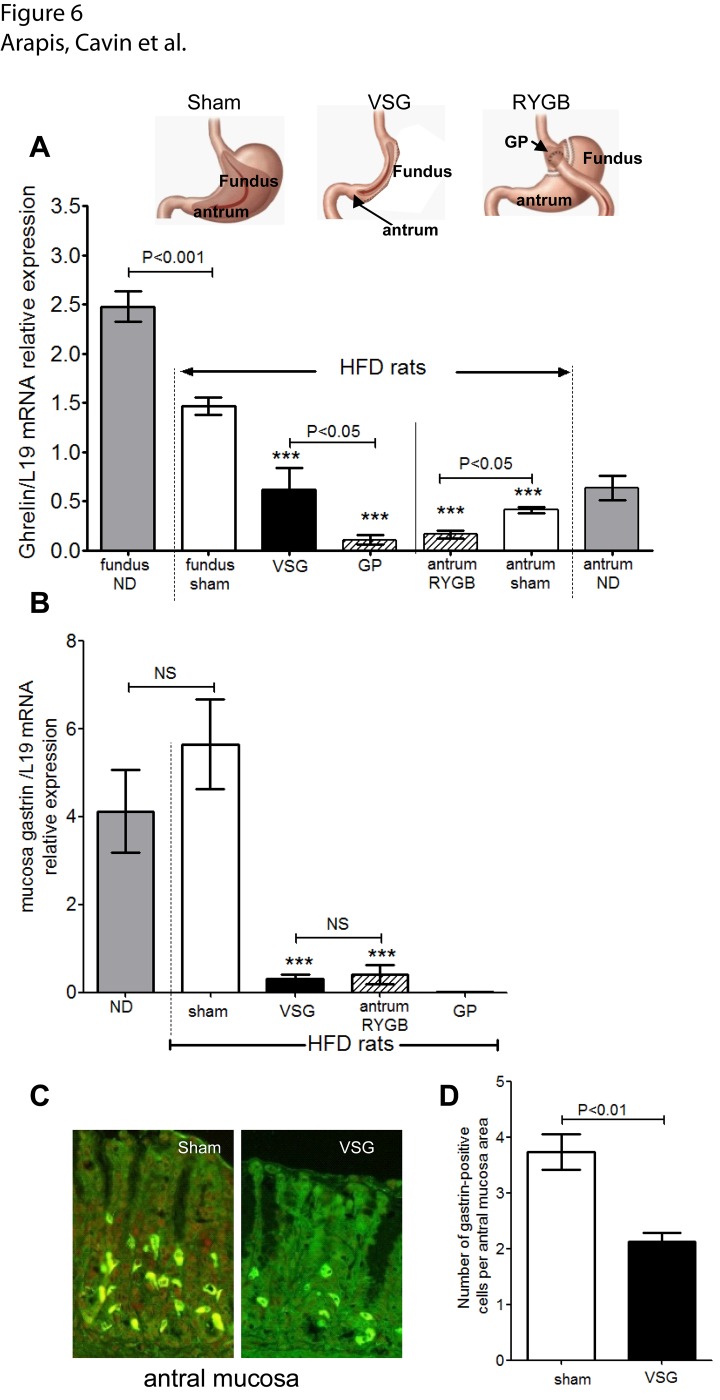
Levels of ghrelin and gastrin mRNA in remodeled stomach after VSG and RYGB. **(A)** *Insert*: *scheme with location of fundus and antrum in normal stomach*, *residual stomach after VSG and gastric pouch (GP) or excluded stomach after RYGB*. **(A)** Quantification of *ghrelin* mRNA in mucosa scrapings of residual fundic mucosa after VSG, fundic mucosa in gastric pouch (GP) after RYGB and antral mucosa of the excluded stomach after RYGB in HFD-obese operated rats in comparison to fundic and antral mucosa from sham-operated ND or HFD rats. **(B)** Quantification of *gastrin* mRNA in mucosa scrapping from antrum of sham-operated ND and HFD rats, antrum of residual stomach after VSG, and antrum of excluded stomach after RYGB. L19 was used as reference. n = 5 rats per group. **(C)** Representative photomicrographs of immunfluorescence of gastrin-positive cells in antral mucosa of sham- and VSG-operated HFD rats. **(D)** Comparative number of gastrin positive cells per antral mucosa area (μm^2^) showing significant decrease in G cell number. Values are means ± SEM for n = 4 in each group.

We addressed the modifications in gastric epithelial cell function comparing fundus and antrum of sham to residual stomach after VSG and RYGB (Fig. 6). HFD fed rats displayed a significant 45% (P<0.001 *vs*. ND) and 30% (P<0.05 *vs*. ND) reduction of *ghrelin* mRNA levels in fundic and antral mucosa, respectively (Fig. 6A). This confirms the impact of the diet on regulation of ghrelin expression.

In the remaining fundic mucosa of VSG-operated obese rats, as soon as 2 weeks post-surgery, *ghrelin* mRNA levels were reduced 2-fold (P<0.01 *vs*. HFD sham fundus) (Fig. 6A). In GP fundic mucosa of RYGB-operated obese rats, a 10-fold reduction (P<0.01 *vs*. HFD sham fundus) of *ghrelin* mRNA levels was observed. Finally, in the excluded stomach of RYGB-operated obese rats, antral mucosa *ghrelin* mRNA levels were reduced by 2-fold (P<0.01 *vs*. sham HFD antrum) strengthening the role of food in the regulation of ghrelin.

We also studied gastrin which is mainly produced by endocrine G cells in antrum of the stomach (Fig. 6B). As shown, *gastrin* mRNA was undetectable in mucosa from GP of RYGB consistent with the well-described absence of G cells in the fundus. In the antral mucosa, *gastrin* mRNA was readily detected and, its levels increased by 20% in the antrum of HFD obese rats compared to ND rats, although this increase was not significant. Moreover, in the antral mucosa of the remaining stomach after VSG, the levels of *gastrin* mRNA were potently reduced (Fig. 6B) consistent with the reduced number of gastrin-positive cells (40% *vs*. sham, P<0.01) (Fig. 6C and D). Finally, *gastrin* mRNA levels were significantly reduced in antral mucosa of the excluded stomach after RYGB. Because the alimentary bolus does not pass through the excluded stomach, these data suggest the importance of food in the physiological regulation of gastrin.

Altogether, these data indicate that VSG and RYGB weight-loss surgeries affect gastric epithelial cell number and function both in fundus and antrum of the remodeled stomach.

## Discussion

Here we provide for the first time, novel and important observations dealing with the remaining gastric mucosa after RYGB and VSG gastrointestinal weight-loss surgeries in HFD obese rat. We report a hyperplasia of the mucous neck cells, a transit cell population of the stomach bearing differentiating capacities into zymogenic or peptic cells.

Bariatric surgery is the most effective treatment for obesity and associated metabolic diseases [1,2,4]. Although several mechanisms have been proposed, the underlying mechanisms of action remain elusive. The development of pre-clinical models of VSG and RYGB have provided valuable tools to gain insights into the endocrine and metabolic mechanisms associated with body-weight reduction and improvement of glucose tolerance [20,21]. In the first reported RYGB rodent model the entire stomach was left intact while the pyloric sphincter was ligated (an experimental model of induction of gastric acid hyper-secretion [28][29,30]. Subsequent models of RYGB were reported and did mimic RYGB surgery in humans with a creation of a true gastric pouch [31–33]. Previous studies suggest that the decline in appetite after VSG or RYGB in morbidly obese individuals was partly due to reduction of plasma ghrelin levels [34,35] however the literature is complex [36] and increase [37,38] or no change [38] in ghrelin levels were also reported after RYGB. We did not measure plasma ghrelin levels, but we found a strong reduction of *ghrelin* mRNA levels in the GP fundic mucosa of RYGB-operated rats and in the residual fundic mucosa after VSG, which is probably due to removal of ghrelin expressing cells mainly located in the fundic mucosa. We also found that VSG- and RYGB-operated animals still lose weight at 5 weeks post-surgery compared to sham whereas all groups have similar calories intake suggesting that appetite was not solely responsible. Thus, our data indicate that gastric ghrelin, even though largely decreased, may not be important for the effects of surgeries in our rat models. Despite quite different surgical restructuration of the gastro-intestinal tract, RYGB and VSG have similar long-term effects on glucose regulation in rats [39,40]. We confirm that RYGB- and VSG-operated animals had different improved oral glucose tolerance, RYGB being more effective than VSG, although both surgical procedures had comparable effects on plasma leptin, glucagon, and insulin levels. These observations confirm that metabolic hormones and weight loss may contribute but are not the only explanation of metabolic amelioration.

The vast majority of previous animal studies have focused on histological examination of intestinal mucosa after RYGB [24,41]. They showed that the Roux and common limbs after RYGB were much heavier and displayed different histological features characterized by increased muscle thickness, mucosal height, villus height and crypt depth [23,24,42,43]. These architectural changes were further shown to be associated, progressively as the gut adapted, with modification in the total number of enteroendocrine cells and their secretion without increased cell density [24,41]. Concerning the residual gastric mucosa, it has been reported that cell proliferation increased and apoptosis was down-regulated in the excluded gastric mucosa of biopsies from RYGB-operated obese patients [44]. In the remaining fundus of the rat after VSG, changes in gastric morphology with gastric foveola elongation, hyperplasia and cystic dilatation of the glands were reported [45]. In our rat model, we did not observe such changes in gastric morphology after VSG but, we found the residual stomach (both fundus and antrum) dilated. This post-operative dilatation of the remaining stomach is comparable with the clinical situation where the stomach was also reported to be dilated after VSG, even after performing a narrow gastric tubulization [46].

Here we report novel observations dealing with the remaining gastric mucosa. Specifically, we present evidence that RYGB and VSG surgeries lead to a new gastric mucosa phenotype characterized by expansion of the mucus neck cells in the oxyntic glands. Interestingly, the MNC are a transit cell population intermediate between gastric stem cells and the differentiated zymogenic cells, which bear the capacity to differentiate into zymogenic or peptic cells (reviewed in ref. [47]). Consistent with previous reports [48,49], these MNC are PAS/BA-positive and thus are able to secrete mucus in the lumen and protect adjacent parietal cells from acid secretion. The expansion of MNC population in the remaining oxyntic mucosa may favor their shift to parietal cells and thus explain the strong immunoreactivity of parietal cell H^+^/K^+^-ATPase. Unexpectedly, and in contrast to residual fundic mucosa after VSG, Ki67-positive proliferating cells were not seen in the GP after RYGB suggesting hyper-differentiation of MNC and differential control of the fundic epithelial cell population. Whether the increased expression of H^+^/K^+^-ATPase β subunit associated with an increased number of parietal cells, correspond to a recovery of acid-secretory capacities of the enlarged parietal cells after VSG and RYGB will be the matter of future studies. We speculate that, after RYGB, in the absence of the duodenum intrinsic acid-buffering properties, the increased parietal cells expressing H^+^/K^+^-ATPase in GP could induce hyperacidity delivered directly in the jejunum lumen that may contribute to anastomotic ulcers (sensitive to proton inhibitors), a recognized complication in some RYGB patients [50,51].

Collectively, these data support the idea that after VSG or RYGB, the remaining gastric mucosa undergoes modification in cell population and function. This is supported by the reduced number of antral gastrin G cells after VSG that correlates with reduced *gastrin* mRNA levels. These data are close to the reported significant decrease of gastrin-positive cells in the antral mucosa of the excluded stomach from RYGB-operated obese patients [44].

In conclusion, this study demonstrates profound changes in the remaining gastric mucosa in terms of differentiation of gastric cell lineages. The findings herein provide new clues for a better understanding of the mechanisms involved in the beneficial effects of bariatric surgery on weight loss and regulation of glucose homeostasis unveiling the importance of the remaining gastric mucosa. A better understanding of the mechanisms by which gastrointestinal weight-loss surgeries induce profound and sustainable effects could facilitate the design of more ideal treatments with maximal effectiveness and minimal invasiveness.

## Supporting Information

S1 Fig Flow diagram of the study. The diets (ND: Normal Diet, HFD: High Fat Diet) received before and after surgery, the division of animals into surgical groups (sham, VSG and RYGB) and the numbers of rats at each time of harvest are presented.
(TIF)

S2 Fig Three-month HFD induces obesity, glucose intolerance and insulin resistance in male Wistar rats. Body-weight curves (**A)** and percent of fat **(B)** and lean mass **(C)** of rats fed ND or HFD for 3 months. Each point represented the mean ± SEM of n = 12 rats for each group. **(D-E)** Changes in blood glucose levels after oral load of glucose (1g/kg BW) **(D)** or intraperitoneal injection of insulin (1U/kg BW) **(E)** in rats fed ND or HFD for 3 months. Each point is the mean ± SEM of n = 9 for the OGTT group and n = 12 for ITT group. Two-Way ANOVA was used to compare body-weight curves, OGTT, and ITT and Mann-Whitney to compare fat and lean mass. **(F)** Representative H&E staining of paraformaldehyde-fixed liver sections from 3-month ND and HFD fed rats. Liver histological analysis revealed that 30% of HFD fed obese rats showed signs of hepatic steatosis with no sign of inflammation or fibrosis. Scale bars correspond to 100μm. Liver *FAS* **(G)** and *ACC* **(H)** mRNA levels in ND and HFD rats. Total RNA was extracted from the liver of ND- and HFD-fed rats. QRT-PCR analysis was performed in duplicate using specific oligonucleotides targeting genes encoding FAS and ACC genes. L19 was used as reference. n = 8 for each group. Mann-Whitney was used to compare the 2 groups.
(TIF)

S3 Fig Time-course of body weight after VSG and RYGB in ND-fed rats. **(A)** VSG- and **(B)** RYGB-induced weight loss in ND fed rats and corresponding sham rats. *Black boxes* correspond to the period of post-operative care and liquid diet consumption before the animals return to free access to solid ND. Results are expressed as percent of loss of body weight over preoperative weight on ND. Two-Way ANOVA was used to compare body-weight curve.
(TIF)

S4 Fig No sign of systemic inflammation 2 weeks after surgery in HFD obese animals. Plasmatic levels of TNF alpha and MCP-1 were assayed 2 weeks after surgery in sham-, VSG- and RYGB-operated animals. n = 2–3 for each group. Kruskal Wallis was used to compare the 3 groups.
(TIF)

S5 Fig Immunostaining of Ki67 proliferating cells. Overview of a representative immunostaining of Ki67 in proliferating cells in formalin-fixed GP anastomosed to jejunal alimentary limb after RYGB. Note that strong Ki67-positive signal was found in proliferating cells of the jejunal crypts *(insert*: *high magnification of jejunum)* while no Ki67-positive cells were detected in the fundic mucosa of the gastric pouch of RYGB.
(TIF)

S1 Table Sequences of primers used for real time quantitative RT-PCR. Gene name and accession number are presented.
(PDF)
