# Supplementary figures and images for: Remodeling of the Residual Gastric Mucosa after Roux-En-Y Gastric Bypass or Vertical Sleeve Gastrectomy in Diet-Induced Obese Rats
Source: PLoS One. 2015 Mar 30;10(3):e0121414. doi: 10.1371/journal.pone.0121414 (PMC4379088; doi:10.1371/journal.pone.0121414)

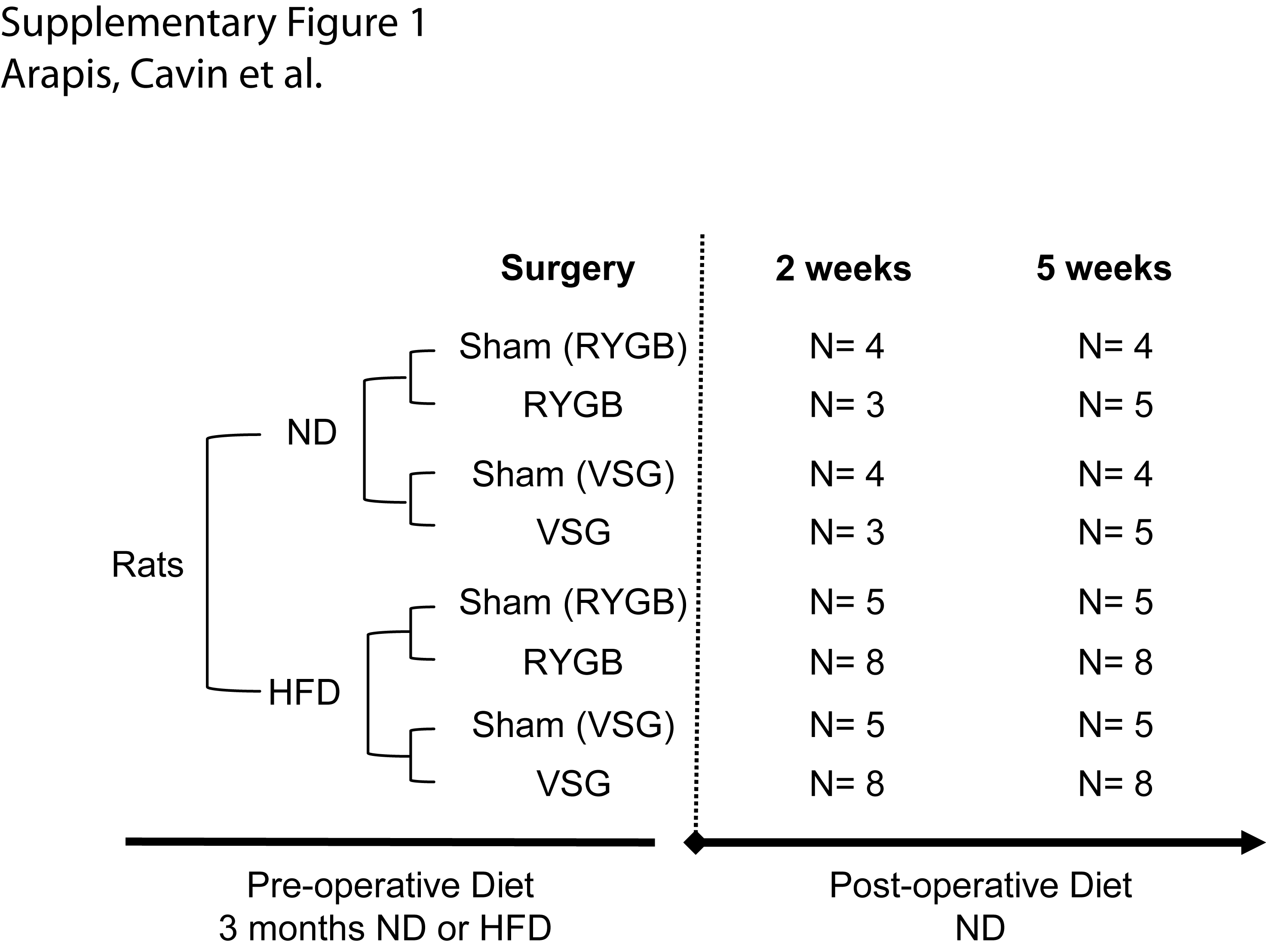

Supplement: S1 Fig — The diets (ND: Normal Diet, HFD: High Fat Diet) received before and after surgery, the division of animals into surgical groups (sham, VSG and RYGB) and the numbers of rats at each time of harvest are presented. (TIF) [file pone.0121414.s001.tif]

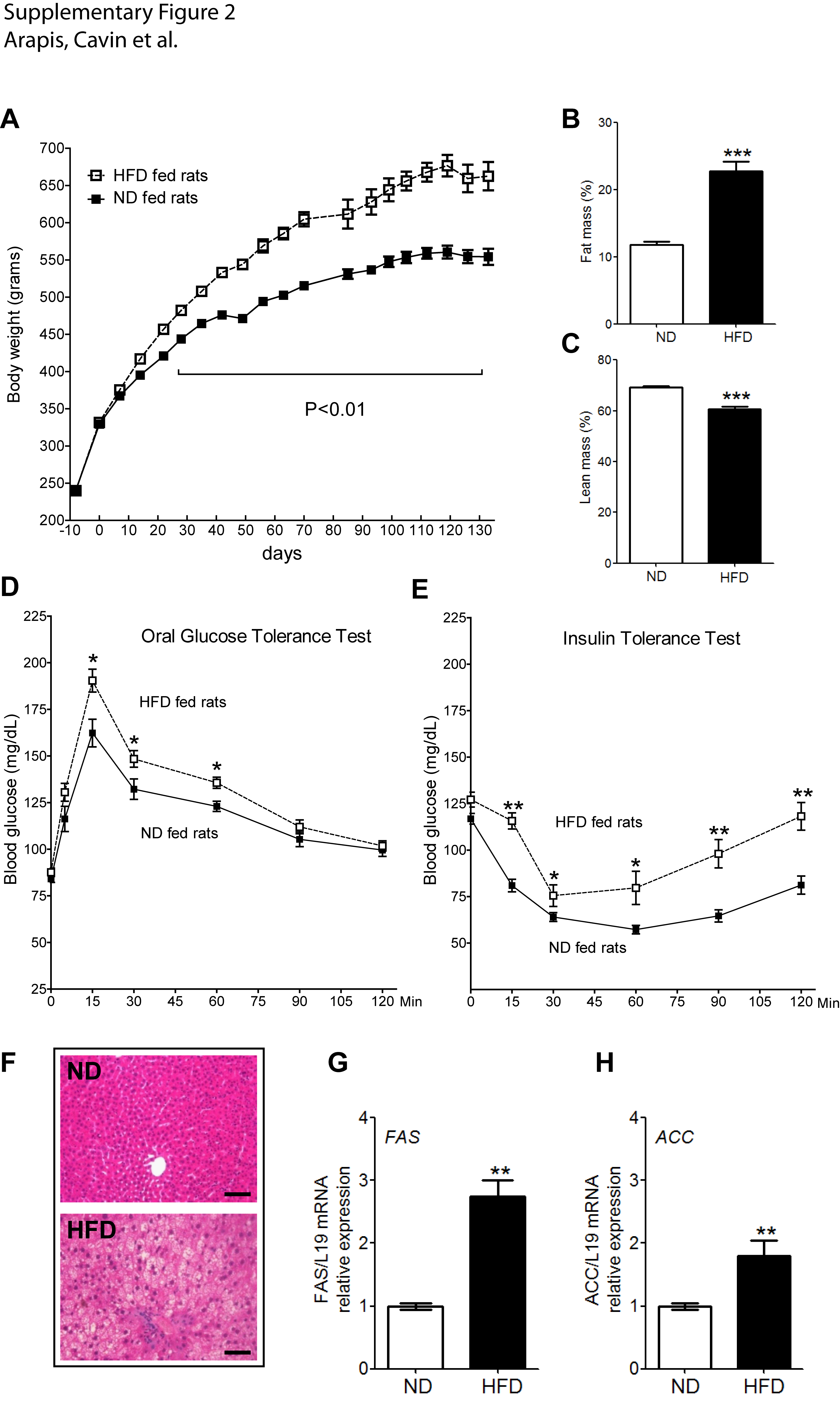

Supplement: S2 Fig — Body-weight curves (A) and percent of fat (B) and lean mass (C) of rats fed ND or HFD for 3 months. Each point represented the mean ± SEM of n = 12 rats for each group. (D-E) Changes in blood glucose levels after oral load of glucose (1g/kg BW) (D) or intraperitoneal injection of insulin (1U/kg BW) (E) in rats fed ND or HFD for 3 months. Each point is the mean ± SEM of n = 9 for the OGTT group and n = 12 for ITT group. Two-Way ANOVA was used to compare body-weight curves, OGTT, and ITT and Mann-Whitney to compare fat and lean mass. (F) Representative H&E staining of paraformaldehyde-fixed liver sections from 3-month ND and HFD fed rats. Liver histological analysis revealed that 30% of HFD fed obese rats showed signs of hepatic steatosis with no sign of inflammation or fibrosis. Scale bars correspond to 100μm. Liver FAS (G) and ACC (H) mRNA levels in ND and HFD rats. Total RNA was extracted from the liver of ND- and HFD-fed rats. QRT-PCR analysis was performed in duplicate using specific oligonucleotides targeting genes encoding FAS and ACC genes. L19 was used as reference. n = 8 for each group. Mann-Whitney was used to compare the 2 groups. (TIF) [file pone.0121414.s002.tif]

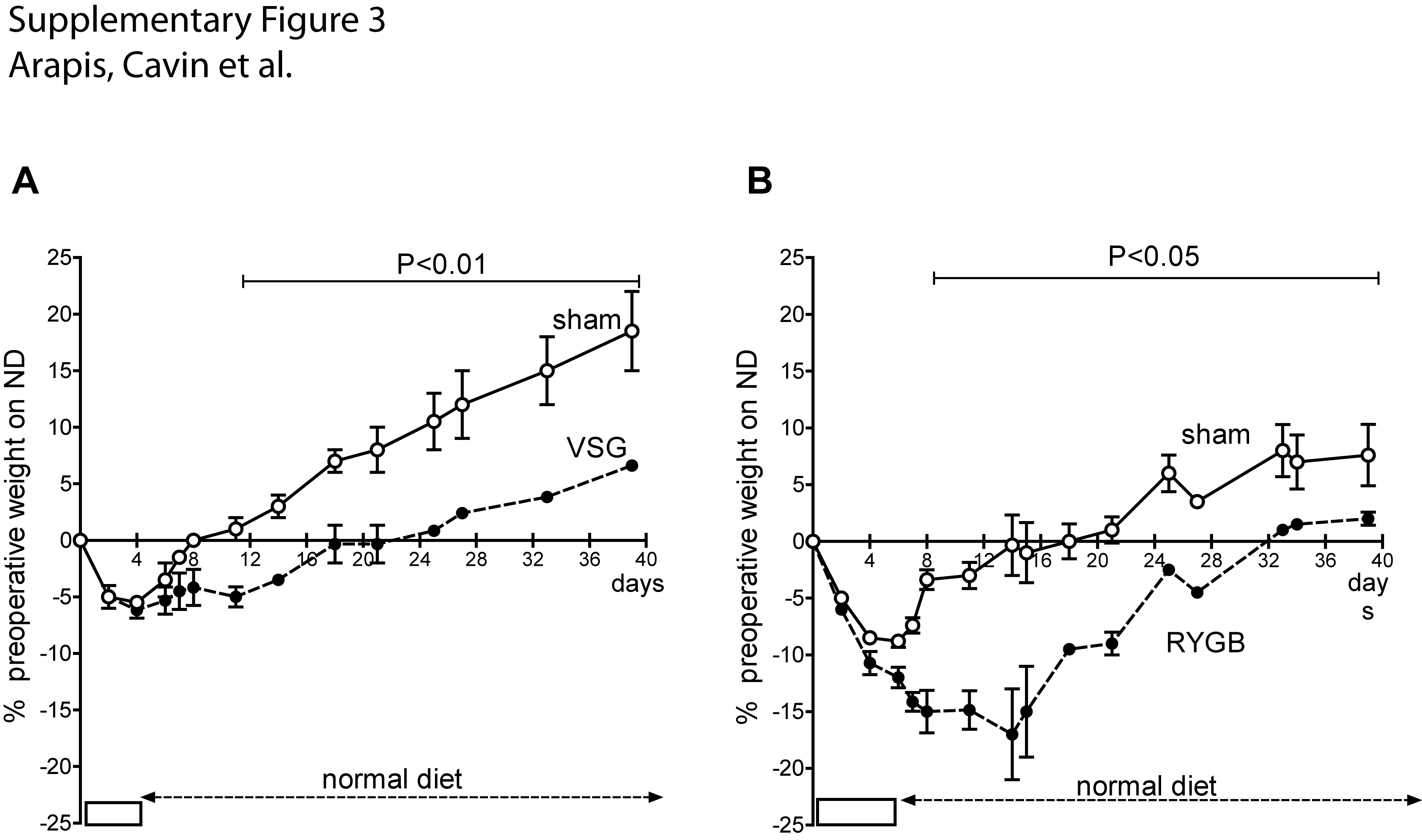

Supplement: S3 Fig — (A) VSG- and (B) RYGB-induced weight loss in ND fed rats and corresponding sham rats. Black boxes correspond to the period of post-operative care and liquid diet consumption before the animals return to free access to solid ND. Results are expressed as percent of loss of body weight over preoperative weight on ND. Two-Way ANOVA was used to compare body-weight curve. (TIF) [file pone.0121414.s003.tif]

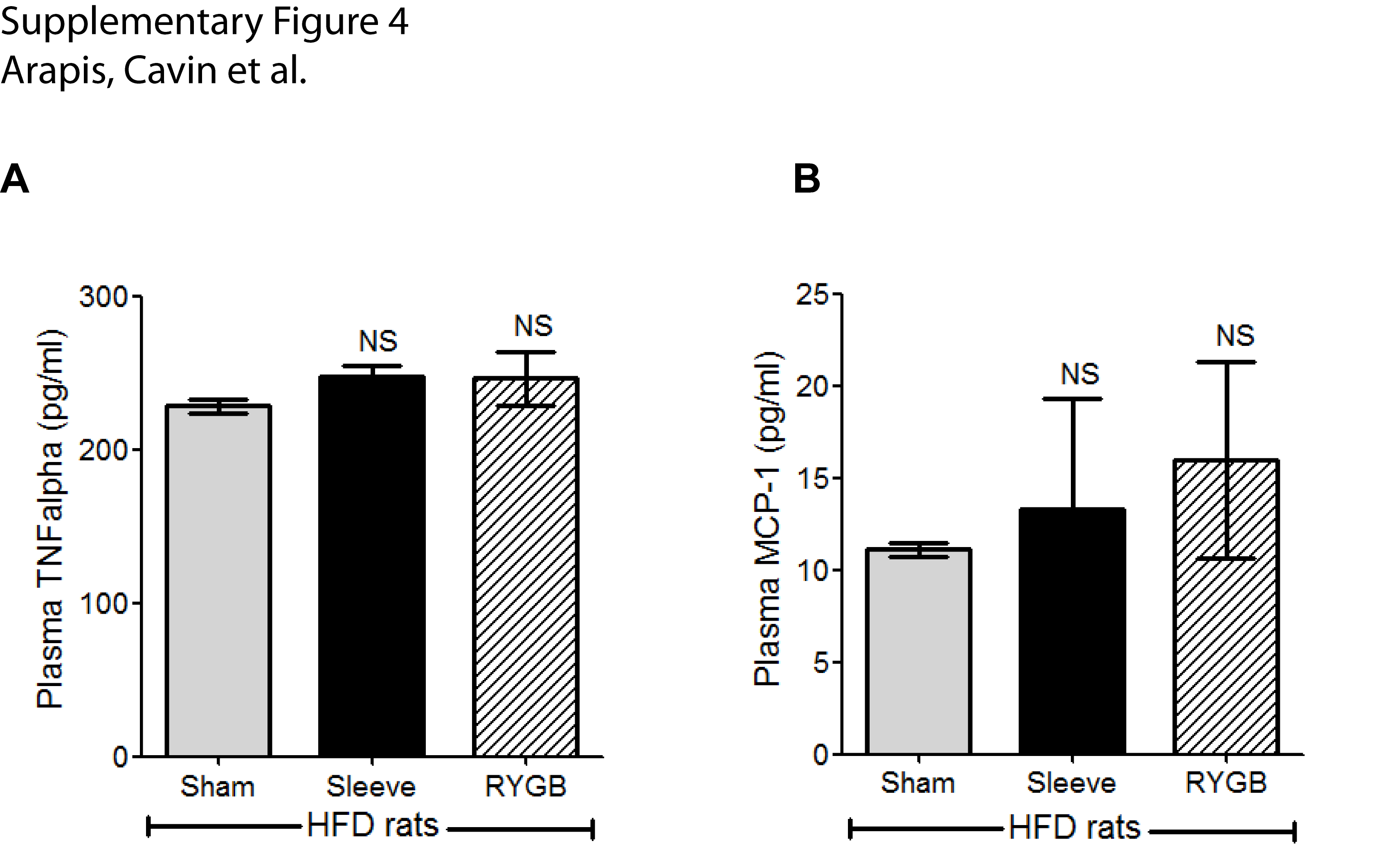

Supplement: S4 Fig — Plasmatic levels of TNF alpha and MCP-1 were assayed 2 weeks after surgery in sham-, VSG- and RYGB-operated animals. n = 2–3 for each group. Kruskal Wallis was used to compare the 3 groups. (TIF) [file pone.0121414.s004.tif]

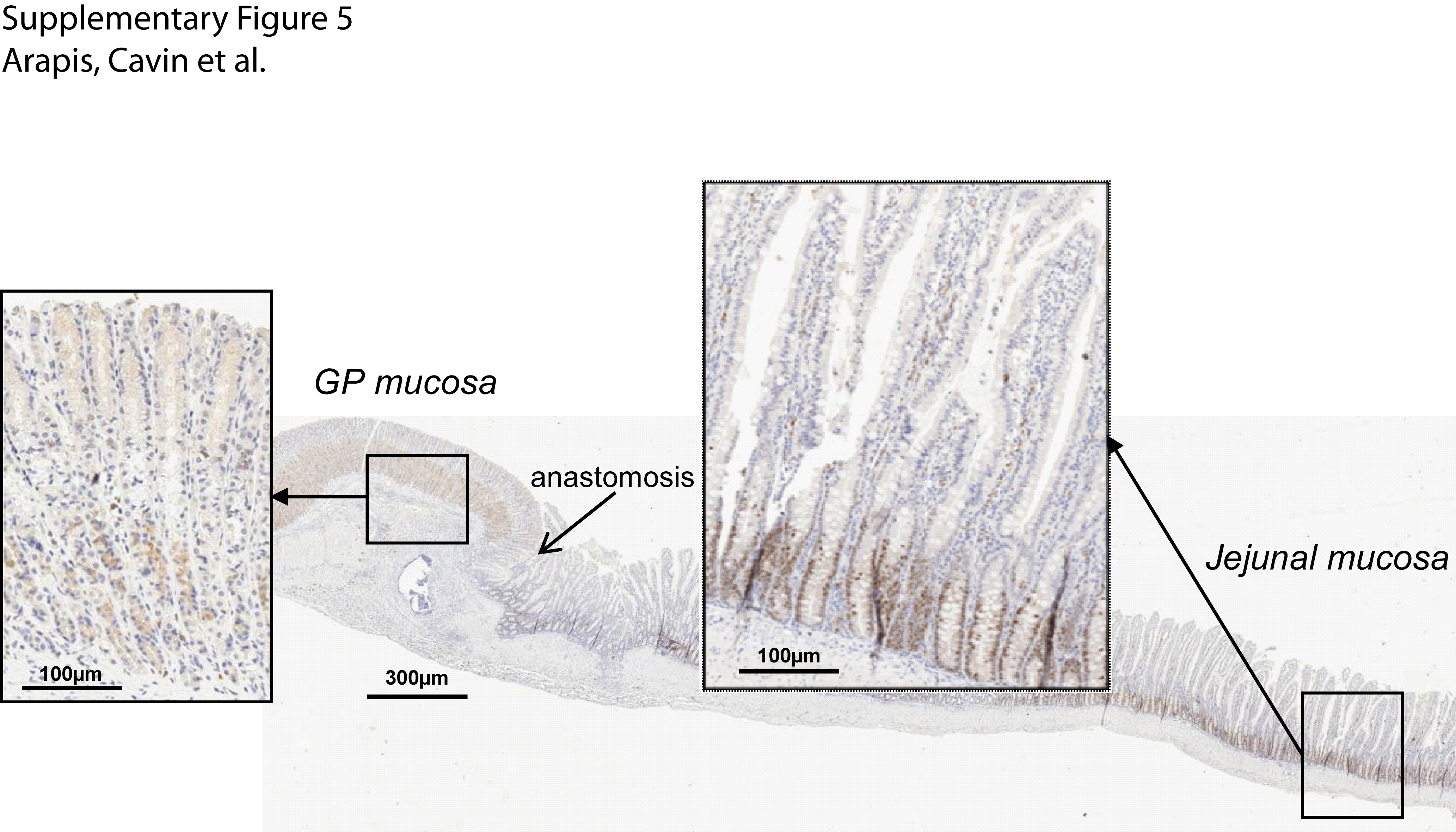

Supplement: S5 Fig — Overview of a representative immunostaining of Ki67 in proliferating cells in formalin-fixed GP anastomosed to jejunal alimentary limb after RYGB. Note that strong Ki67-positive signal was found in proliferating cells of the jejunal crypts (insert: high magnification of jejunum) while no Ki67-positive cells were detected in the fundic mucosa of the gastric pouch of RYGB. (TIF) [file pone.0121414.s005.tif]
