# Supplementary material for: Remodeling of the Residual Gastric Mucosa after Roux-En-Y Gastric Bypass or Vertical Sleeve Gastrectomy in Diet-Induced Obese Rats
Source: PLoS One. 2015 Mar 30;10(3):e0121414. doi: 10.1371/journal.pone.0121414 (PMC4379088; doi:10.1371/journal.pone.0121414)
Supplement: S1 Table — Gene name and accession number are presented. (PDF) [file pone.0121414.s006.pdf]

**Gene**	**Accession**	**Primers** (5'->3')
L19	NM_031103.1	Forward TGCCGGAAGAACACCTTG<br>Reverse GCAGGATCCTCATCCTTCG
Ghrelin	NM_021669.2	Forward CCCAGAGGACAGAGGACAAG<br>Reverse AACATCGAAGGGAGCATTGA
Gastrin	NM_012849.1	Forward CCGCAACACTTCATAGCAGA<br>Reverse CATCCATCCGTATGCTTCCT
FAS	NM_017332.1	Forward GGCCACCTCAGTCCTGTTAT<br>Reverse AGGGTCCAGCTAGAGGGTACA
ACCalpha	NM_022193.1	Forward ACAGAGATGGTGGCTGATGTC<br>Reverse GATCCCCATGGCAATCTG
